# Free Fatty Acids Modulate Thrombin Mediated Fibrin Generation Resulting in Less Stable Clots

**DOI:** 10.1371/journal.pone.0167806

**Published:** 2016-12-12

**Authors:** Anna Tanka-Salamon, Erzsébet Komorowicz, László Szabó, Kiril Tenekedjiev, Krasimir Kolev

**Affiliations:** 1 Department of Medical Biochemistry, Semmelweis University, Budapest, Hungary; 2 IMEC, Research Centre for Natural Sciences, Hungarian Academy of Sciences, Budapest, Hungary; 3 Nikola Vaptsarov Naval Academy, Varna, Bulgaria; 4 Australian Maritime College, University of Tasmania, Newnham, Australia; GERMANY

## Abstract

Upon platelet activation, free fatty acids are released at the stage of thrombus formation, but their effects on fibrin formation are largely unexplored. Our objective was *t*o characterize the kinetic effects of fatty acids on thrombin activity, as well as the structural and mechanical properties of the resultant fibrin clots. Thrombin activity on fibrinogen was followed by turbidimetry and detailed kinetic characterization was performed using a fluorogenic short peptide substrate. The viscoelastic properties of fibrin were measured with rotatory oscillation rheometer, whereas its structure was analyzed with scanning electron microscopy (SEM). In turbidimetric assays of fibrin generation, oleate and stearate at physiologically relevant concentrations (60–600 μM) produced a bell-shaped inhibitory dose response, increasing 10- to 30-fold the time to half-maximal clotting. Oleate inhibited thrombin activity on a short peptide substrate according to a mixed-type inhibitor pattern (a 9-fold increase of the Michaelis constant, *K*_*m*_ and a 20% decrease of the catalytic constant), whereas stearate resulted in only a minor (15%) drop in the catalytic constant without any change in the *K*_*m*_. Morphometric analysis of SEM images showed a 73% increase in the median fiber diameter in the presence of stearate and a 20% decrease in the presence of oleate. Concerning the viscoelastic parameters of the clots, storage and loss moduli, maximal viscosity and critical shear stress decreased by 32–65% in the presence of oleate or stearate, but loss tangent did not change indicating decreased rigidity, higher deformability and decreased internal resistance to shear stress. Our study provides evidence that free fatty acids (at concentrations comparable to those reported in thrombi) reduce the mechanical stability of fibrin through modulation of thrombin activity and the pattern of fibrin assembly.

## Introduction

Atherothrombotic lesions of stenosed intracranial and extracranial vessels may lead to microembolization resulting in multiple small cerebral infarcts and progressive cognitive impairment [1–3]. Moreover, microembolization is reported to be the ultimate cause of myonecrosis in patients dying of acute coronary thrombosis [4]. Peripherial occlusions in the extremities, mesenteric or renal arteries due to arterial embolism may also have serious consequences such as ulceration, gangrene, amputation or even death [5,6]. Despite the efforts made in the last two decades to explore previously unknown mechanisms of thrombus formation and to characterize the resultant clot stability (reviewed in [7,8]), our understanding of the non-conventional determinants of the structure and mechanical stability of thrombi is still far from being complete.

In addition to the release of a broad spectrum of hemostatic proteins and signal molecules during platelet activation, the increase in the cytosolic calcium concentration leads to activation of the cytosolic phospholipase-A_2_. This enzyme hydrolyses membrane phospholipids releasing free fatty acids and lysophospholipid [9]. The high degree of platelet compaction in arterial thrombi [10], and the activation-dependent lipidomic flux in platelets [11], result in accumulation of phospholipids and fatty acids at concentrations in the millimolar range [12]. The major phospholipid in platelet membranes is lecithin and more than 40% of its total fatty acid content is represented by oleic (18:1) and stearic (18:0) acid [13]. Thus, the remodeling of phospholipids within thrombi raises the concentrations of oleic and stearic acid above their plasma levels (180 and 70 μM, respectively [14]). Being present both at the clotting phase and the breakdown of thrombi, free fatty acids are potential modulators of thrombus formation and thrombolysis. The presence of fatty acids in the clot is known to directly affect the activity of proteases involved in fibrin degradation [12,15,16], but their effects on the enzyme activities in fibrin formation (e.g. thrombin) and the resultant clot structure and stability have hardly been investigated.

Variations in fatty acid content at the level of the whole body are known to affect the procoagulant mechanisms. Recent reports evidence reduced thrombin generation after consumption of fatty acids [17–19]. Inhibition of thrombin by oleic and stearic acid has been reported in amidolytic assays [20]. Furthermore, fatty acid esters of polyphenols are more efficient inhibitors of thrombin, than free polyphenols [21]. Hindrance of thrombin activity occurs mostly through noncovalent interactions, as demonstrated for several natural [22–24] and synthetic [25] thrombin inhibitors, however feasible molecular interactions between free fatty acids and thrombin as well as the exact kinetic characteristics of thrombin-mediated clot formation in the presence of these modulators are scarcely explored. The possibility of a noncovalent interaction between free fatty acids and fibrinogen has also emerged, since molecules forming hydrogen bonds with fibrinogen, may alter the structure of the fibrin clots due to the major role of H-bonds in the polymerization step of fibrin clot formation [26].

The overall aim of this study was to characterize the kinetic effects of free fatty acids on thrombin activity and also the structural and mechanical properties of the formed clots. Our study revealed that free oleic and stearic acids at biologically relevant concentrations modify the kinetics of fibrin formation and the ultrastructure of the fibrin network, resulting in clots that can be mechanically disassembled at shear stress of magnitude corresponding to the hydrodynamic conditions in partially occluded coronaries [27]. The impaired mechanical stability of fibrin suggests that the free fatty acid content of arterial thrombi [12] could contribute to the risk of microembolization *in vivo*.

## Materials and Methods

### Proteins and reagents

If not otherwise indicated, experiments were performed in HEPES buffered saline (HBS, 10 mM HEPES, 150 mM NaCl, pH 7.4) using bovine thrombin, purchased form Serva Electrophoresis GmbH (Heidelberg, Germany) and further purified as described in [28] yielding a preparation with specific activity of 2100 IU/mg [29]. Thrombin activity of 1 IU/mL was considered equivalent to approximately 10.7 nM by active site titration [30]. Human thrombin (01/580 the WHO 2^nd^ International Standard for alpha thrombin) was obtained from NIBSC (South Mimms, UK). Sodium salt of oleic and stearic acids were from Sigma-Aldrich Kft. (Budapest, Hungary) and stock solutions (10 mM) were prepared in water (prewarmed to 70°C) containing 50 μM butylated hydroxytoluene. These stock solutions were further diluted to the desired concentrations in HBS. (At the final concentration in the reaction mixtures butylated hydroxytoluene had no effect on the thrombin activity on its own). Fibrinogen (human, plasminogen-depleted) was from Calbiochem (San Diego, CA, USA) and fluorogenic thrombin substrate butoxycarbonyl-Val-Pro-Arg-7-amido-4-methylcoumarin (Boc-VPR-AMC) was from R&D Systems (Minneapolis, MN, USA).

### Kinetics of fibrin formation by thrombin

Turbidimetric assays were performed to investigate the effect of free fatty acids on the kinetics of fibrinogen clotting by thrombin. Fibrinogen at 7.5 μM was clotted with 20 nM thrombin in the presence of 20–800 μM sodium oleate or 25–1500 μM sodium stearate in microplate wells at 37°C. The course of clot formation was monitored by measuring the light attenuation at 340 nm with a Zenyth 200rt microplate spectrophotometer (Anthos Labtec Instruments GmbH, Salzburg, Austria) and T_50_ values (the time needed to reach the half maximal turbidity) were determined.

### Kinetics of short peptide hydrolysis by thrombin

Because turbidimetric measurements generate a combined signal that reflects the outcome of fibrinogen cleavage by thrombin and the polymerization of fibrin monomers, an alternative fluorometric assay on the substrate Boc-VPR-AMC was used to directly characterize the effect of free fatty acids on the catalytic activity of thrombin. Following preliminary estimates of the Michaelis constant (*K*_*m*_) for each modulator concentration, hydrolysis of Boc-VPR-AMC (at six different concentrations ranging from 0.5*K*_*m*_ to 5*K*_*m*_) by 10 nM thrombin was monitored in the presence of 0; 50; 100 or 200 μM sodium oleate or stearate at 37°C. In some cases 100 μM bovine serum albumin was added to the reaction mixture as a positive control. Fluorescence intensity (which reflects the release of amido-methylcoumarin) was measured continuously for 80 s with a CLARIOstar® microplate reader (BMG LABTECH GmbH, Ortenberg, Germany) (excitation: 380 nm, emission: 460 nm). The delay time between the initiation of the reaction and the first measurement point was estimated with linear extrapolation from the initial six measured RFU (Relative Fluorescence Units) values back to baseline fluorescence, and a coefficient of 262.7 RFU μM^-1^ cm^-1^ (determined from calibration in our assay system) was used to convert the measured fluorescence values to product concentration. The kinetic parameters of thrombin were estimated according to the following model mechanism $E+S\overset{k_{1}}{\underset{k_{-1}}{⇄}}ES\overset{k_{2}}{\to }E+P$, where *E* is thrombin, *S* is Boc-VPR-AMC, *P* is amido-methylcoumarin and *k*_*1*_, *k*_*2*_ and *k*_*-1*_ are the respective reaction rate constants. With the quasi-steady-state assumption the differential rate equation for this scheme is

$$\frac{dP}{dt}=\frac{k_{p}.E_{t0}.(S_{0}-P)}{K_{m}+S_{0}-P},$$ (Eq. 1)

where *E*_*t0*_ and *S*_*0*_ are the initial concentrations of thrombin and its substrate, the Michaelis constant *K*_*m*_ *= (k*_*-1*_*+k*_*2*_*)/k*_*1*_ and the catalytic constant *k*_*p*_ *= k*_*2*_ [15].

A previously described numerical procedure [15] including generation of 1000 synthetic sample sets for each experimental setting and Monte Carlo simulation of the reaction progress curves was applied to identify the final best estimates and the 95% ‘root’ confidence intervals of the catalytic constant (*k*_*p*_) and *K*_*m*_ according to the integrated form of Eq 1:

$$t=\frac{1}{k_{p}.E_{t0}}P+\frac{K_{m}}{k_{p}.E_{t0}}\ln\frac{S_{0}}{S_{0}-P}.$$ (Eq. 2)

The abovementioned numerical evaluation comprises a table look-up procedure instead of the regression analysis of the linearized version of Eq 2. All calculations were performed in Matlab R2016a (The MathWorks, Inc., Natick, MA, US).

### Rheological Measurements of Fibrin Clots

The effects of free fatty acids on the viscoelastic properties of fibrin clots were studied in a cone-and-plate type oscillation rheometer (HAAKE RheoStress 1, Thermo Scientific, Karlsruhe, Germany). Fibrinogen at 6 μM, premixed with 100 μM sodium oleate or stearate was clotted with 10 nM thrombin in the measurement gap of the rheometer at 37°C. An oscillatory strain (1 Hz, 0.015 strain amplitude) was imposed on the samples, and viscoelastic parameters (storage modulus, *G’* and loss modulus, *G”*) were recorded with the help of the HAAKE RheoWin v. 3.50.0012 data manager software (Thermo Scientific) for 10 min, a time interval sufficient to establish the trend of the measured parameters in all samples. For determining the gel/fluid transition of the same clots, a stepwise increasing shear stress (*τ*) of 0.01 to 1000 Pa was applied and dynamic viscosity (*η*) was determined at each step. For numerical description of flow curves, maximal viscosity (*η*_*max*_) and critical shear stress values (*τ*_*flow*_, at which viscosity falls to zero and gel/fluid transition occurs) were determined. The statistical significance of differences in the parameters was evaluated with the Kolmogorov-Smirnov test at *p<*0.05 level.

### Scanning Electron Microscope Imaging of Fibrin Clots

To examine the effect of free fatty acids on the structure of fibrin, 7.5 μM fibrinogen was clotted with 20 nM thrombin for 3 hours at 37°C in the presence of 40–400 μM sodium oleate or 50–500 μM sodium stearate. The fibrin clots were fixed in 1%(v/v) glutaraldehyde in 100 mM sodium cacodylate, pH 7.2, dehydrated in a series of ethanol dilutions, ethanol/acetone and pure acetone followed by critical point drying with CO_2_ in an E3000 Critical Point Drying Apparatus (Quorum Technologies, Newhaven, UK). The specimens were mounted on adhesive carbon discs, sputter-coated with gold in an SC7620 Sputter Coater (Quorum Technologies), and images were taken with a scanning electron microscope (SEM) EVO40 (Carl Zeiss GmbH, Oberkochen, Germany). Fibrin fiber diameters were measured and data distributions were analyzed using Kuiper’s test and Monte Carlo simulation procedures running under Matlab R2016a [31,32].

### Particle Size Determination by Dynamic Light Scattering

The particle size distribution profile of the sodium oleate and stearate solutions used in the above-mentioned experiments (0; 200; 400; 800 or 1200 μM sodium oleate or stearate in the presence of 7.5 μM fibrinogen in HBS) was measured with a W130i dynamic light scattering system (AvidNano, London, UK). Measurement data analysis including determination of the polydispersity index (PDI) was performed with the i-Size software, supplied with the system by the manufacturer. PDI is the square of the light scattering polydispersity (the ratio of absolute width and mean value of the size distribution) of dissolved particles. A PDI value of 0 refers to a monodisperse system with uniform particles, while a PDI of >0.4 suggests a polydisperse system with particle sizes varying too widely for exact size discrimination.

## SDS Polyacrylamide Gel Electrophoresis of Thrombin Autodigestion Products

To investigate the impact of free fatty acids on the process of thrombin autocleavage, as an indicator of molecular interactions between the enzyme and the modulators, human thrombin at 6 μM was incubated with 0 or 1 mM of sodium oleate or stearate at 37°C for 72 hours. Samples were treated with non-reducing denaturing buffer (50 mM Tris-HCl, 50 mM NaCl, 2% (w/v) SDS, pH 8.2) and heated at 95°C for 3 min. Gel electrophoresis was performed on a 10–15% (w/v) gradient polyacrylamide gel, followed by visualization of protein bands with silver staining.

## Results

### Effects of free fatty acids on thrombin activity

**Fig 1:**
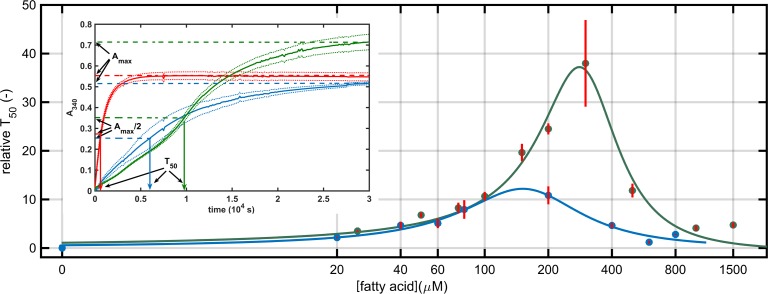
Effect of free fatty acids on the kinetics of thrombin-mediated fibrin generation. Fibrinogen was clotted with thrombin in the presence of sodium oleate or stearate. The course of clot formation was monitored by measuring the absorbance at 340 nm and T_50_ was defined as the time needed to reach the half-maximal turbidity (illustrated in the Inset for clotting in the absence of any additive in red, in the presence of 200 μM stearate in green or oleate in blue as mean with continuous lines ± 1SEM with dotted lines, *n* = 5). Relative T_50_ values are presented in green for stearate and blue for oleate (T_50_ measured in the absence of additives is considered to be 1) as mean (symbols) and SEM (red bars), n = 5. Lines represent the optimal fit to a ratio of empirical polynomial functions with a degree of 2 for the power coefficient in both the numerator and denominator functions (Curve Fitting Tool 3.5.3 of Matlab 2016a).

Two different kinetic assays were applied to investigate the influence of free fatty acids on thrombin activity. A turbidimetric assay was performed to monitor the action of thrombin on its natural substrate, fibrinogen in the presence of sodium salts of fatty acids. The increase of absorbance in this assay reflects the formation of polymerizing fibrin, the final structure of which causes variations in the absolute maximal values of absorbance. In this assay the time to reach half-maximal turbidity (T_50_) can be used to describe the kinetics of fibrin formation, because its values do not depend on these structural variations of the final clots. We found that both oleate and stearate delayed the clotting process and produced a bell-shaped inhibitory dose response of thrombin-mediated fibrin generation. Stearate had a stronger impact on the rate of clot formation: T_50_ increased 38-fold at the most effective modulator concentration (300 μM), while oleate caused up to a 12-fold increase in the T_50_ values (Fig 1).

**Fig 2:**
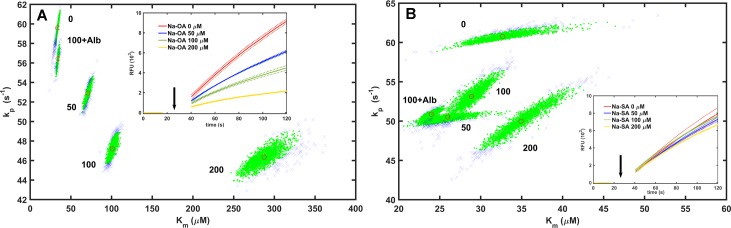
Effect of free fatty acids on the kinetic parameters of thrombin on a small peptide- substrate. Boc-VPR-AMC at 6 different concentrations adjusted to match the range of 0.5*K*_*m*_ to 5*K*_*m*_ for each modulator concentration was added to thrombin and different concentrations of sodium oleate (A) or stearate (B) (three parallel series of each substrate concentrations were measured for each fatty acid concentration). The release of amido-methylcoumarin was measured continuously and the progress curves were analyzed to estimate the kinetic parameters (*k*_*p*_, *K*_*m*_) according to the procedures described in the Materials and methods. The estimated synthetic parameter pairs within the 95% confidence region are shown by green symbols and the pairs out of this region are presented in blue (the numbers indicate the concentration of the fatty acid in μM, for which the respective data set was generated in the Monte-Carlo simulation, +Alb indicates the presence of 100 μM albumin). The best estimate from the Monte-Carlo simulation is indicated by a red circle. The exact numerical values are presented in Table 1. Insets show progress curves (mean with continuous lines ± 1SEM with dotted lines, *n* = 3) at 80 μM Boc-VPR-AMC and different sodium stearate or oleate concentrations (the arrows indicate the initiation of the reaction with thrombin injection into the substrate).

In the second kinetic assay, thrombin activity was measured on a fluorogenic peptide substrate, which allowed a detailed analysis of the kinetic parameters (*k*_*p*_, *K*_*m*_) of thrombin in the presence of fatty acids. In this experimental setup stearate was found to have no biologically relevant effect on the kinetic parameters, whereas oleate exhibited a mixed-type inhibitory effect on the enzyme, decreasing *k*_*p*_ from 59.67 (57.85–61.41) to 46.23 (43.83–49.04) s^-1^ and increasing *K*_*m*_ from 33.88 (31.07–36.84) to 285.80 (246.98–332.42) μM (best estimates and their 95% ‘root’ confidence intervals) (Fig 2, Table 1). Equimolar concentrations of albumin that binds the free fatty acids completely abrogate the inhibiting effect of oleate (Table 1).

**Table 1:** Kinetic parameters of thrombin in the presence of free fatty acids.

	oleate (μM)						stearate (μM)					
	0		50	100		200	0		50	100		200
		+Alb			+Alb			+Alb			+Alb	
***K***_***m***_ **(μM)**	**33.9**	**45.6**	**70.8**	**100.8**	**33.9**	**285.8**	**32.5**	**32.0**	**25.5**	**28.9**	**24.0**	**34.9**
**95% ‘root’ CI**	31.1–36.8	43.3–48.2	63.5–78.6	92.6–111.0	31.7–36.2	246.9–332.4	24.7–46.4	30.0–34.4	20.6–33. 6	25.0–32.9	22.0–25.9	29.3–42. 6
***k***_***p***_ **(s**^**-1**^**)**	**59.7**	**66.1**	**52.9**	**47.2**	**56.4**	**46.2**	**60.7**	**68.3**	**50.3**	**53.2**	**50.9**	**50.0**
**95% ‘root’ CI**	57.9–61.4	64.7–67.5	50.9–54.7	45.7–48. 9	54.6–58.1	43.8–49.0	58.9–62.6	66.7–69.7	49.3–51.3	50.1–56.4	49.8–51.9	46.3–54.4

Numerical values for the Michaelis constant (*K*_*m*_) and the catalytic constant (*k*_*p*_) of thrombin on Boc-VPR-AMC substrate were determined as illustrated in Fig 2 and are presented as best estimates and their confidence intervals (CI) from Monte-Carlo simulation of 1000 cycles performed as described in Materials and methods. The presence of 100 μM albumin is indicated as +Alb.

**Fig 3:**
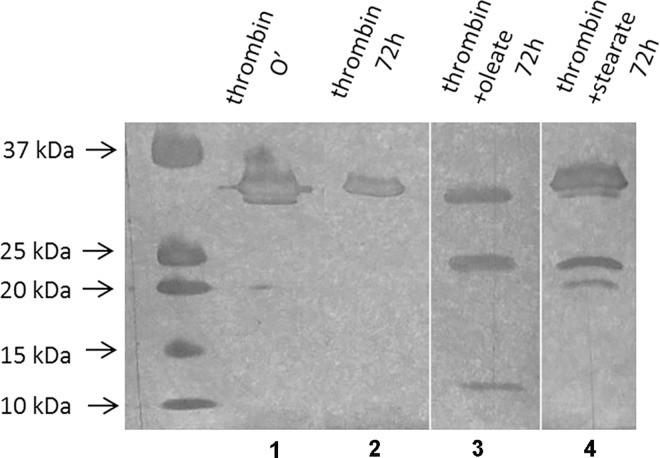
Effect of free fatty acids on thrombin autodigestion. Thrombin was incubated in the presence or absence of sodium oleate or stearate at 37°C for 72 hours. Samples were treated with a non-reducing denaturing buffer and polyacrylamide gel electrophoresis was performed, followed by visualization of protein bands by silver staining. Samples run on different gels are separated by vertical white lines. Lanes 1 and 2: Thrombin incubated in the absence of modulators for 0 min and 72 hours. Lanes 3 and 4: Thrombin incubated in the presence of oleate or stearate for 72 hours.

To address the mechanism of these inhibiting effects, the autodigestion of thrombin was examined in the presence and absence of fatty acids. In the absence of additives gel electrophoresis did not detect any autodigestion during the first 72 hours, whereas oleate and stearate both accelerated the self-destroying enzymatic process but with a different size profile of the degradation products (Fig 3). Differences in the band sizes in the case of oleate and stearate are attributable to ongoing autocleavage of presumably different mechanisms.

Because in our experimental setup fatty acids are exposed to pH below their pK_a_ values (10.15 for stearic- and 9.85 for oleic acid) [33], most probably they participate in the form of aggregates as suggested by the results of the dynamic light scattering measurements evidencing extreme polydispersity (PDI>2) for fibrinogen-fatty acid-HBS solutions. Exact particle size determination could not be performed, since PDI values over 0.4 refer to an extremely broad size distribution. Sodium salts of fatty acids give a transparent, colorless solution when dissolved in water containing butylated hydroxytoluene at 37°C. However, when diluted in HBS at pH 7.4, their solution turns opalescent indicating aggregate or microcrystal formation. The opalescence is more intense in the case of stearate in line with its melting point of 70°C, much higher than that of oleic acid (13–14°C).

### Effects of free fatty acids on the structural and mechanical properties of fibrin clots

**Fig 4:**
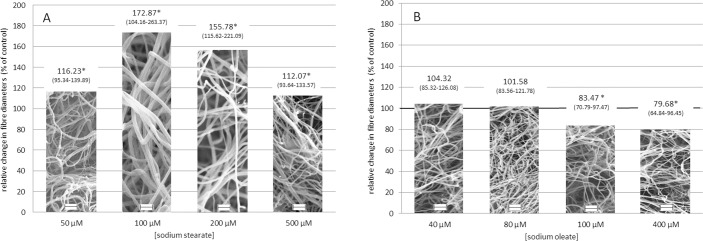
Effect of free fatty acids on fibrin fiber diameters. Fibrinogen premixed with sodium stearate (A) or oleate (B) at the indicated concentrations was clotted with thrombin. Clots were processed for SEM imaging and fibrin fiber diameters were measured and analyzed as detailed in Materials and methods. Bars contain representative images of fibrin with the indicated additive (scale bar = 1 μm) and the median values (bottom—top quartiles) of the diameter distributions are shown above each bar. The height of the bars indicates the relative change in fiber diameter as percentage of the median values in the absence of additives. Asterisk indicates statistical significance at p<0.001 according to Kuiper’s test in comparison to pure fibrin.

Because fibrin turnover *in vivo* depends on the lytic susceptibility and mechanical stability of the clots and these properties are directly affected by the three-dimensional structure and viscoelastic characteristics of fibrin [28,31,34,35] and reviewed in [36, 37], it was of interest to investigate the impact of fatty acids on the structure and mechanical stability of fibrin. Morphometric analysis of SEM images showed significant fiber thickening at 100–200 μM of stearate, with a maximum increase of the median diameter by 73% (from 50.5 to 87.3 nm), while fiber diameter values approximated control levels at 500 μM stearate yielding a bell-shaped dose-dependence, similar to the dose-response curve in Fig 1. Oleate had a weak thinning effect producing a 20% drop in median fiber diameter at 400 μM oleate (Fig 4).

**Fig 5:**
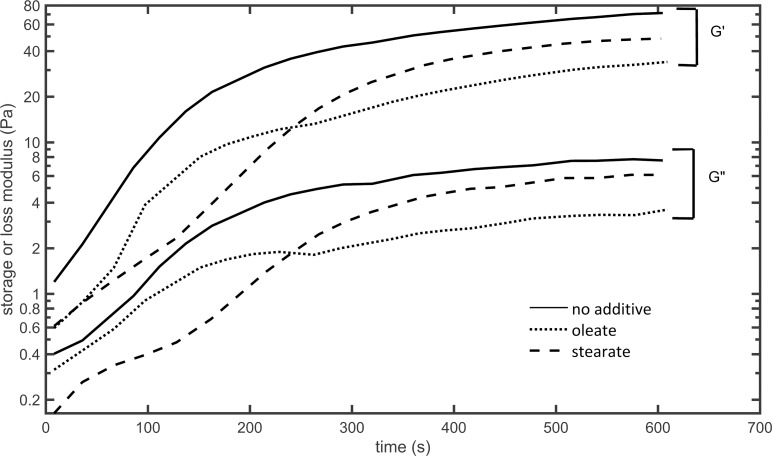
Effect of free fatty acids on the course of rigidity changes during clot formation. Fibrinogen premixed with sodium oleate or stearate was clotted with thrombin in the measurement gap of a cone-and-plate type oscillation rheometer and an oscillatory strain was imposed on the samples. Representative curves of storage (G') and loss (G'') moduli for each clot type are presented, all rheological parameters with statistics are shown in Table 2.

**Fig 6:**
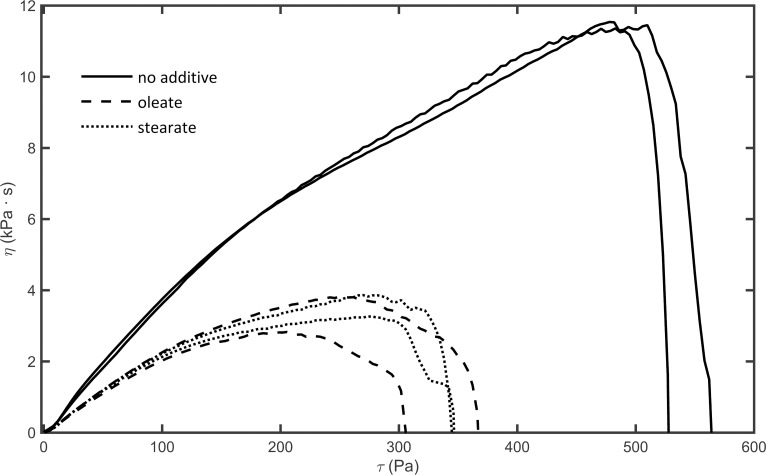
Effect of free fatty acids on the gel/fluid transition of fibrin clots. Fibrin clots were prepared in the measurement gap of a cone-and-plate type oscillation rheometer as detailed in Materials and methods. Thereafter, stepwise increasing shear stress (*τ*) was applied to the clot and dynamic viscosity (*η*) was determined. The abrupt fall in viscosity to 0 indicates the gel/fluid transition of the fibrin clots (*τ*_*flow*_ in Table 2). Two representative curves are shown for each clot type.

The viscoelastic properties of the clots were determined by oscillatory rheometry. In the course of fibrin polymerization, the storage (G’) and loss (G”) moduli of the samples increased over time (Fig 5). In line with the turbidity data, the rheometry measurements evidenced slower clotting and lower values of both storage and loss moduli in the presence of fatty acids, while the loss tangent (G”/G’) remained unchanged (Table 2). When increasing shear stress was imposed upon completely polymerized clots, critical shear stress values (*τ*_*flow*_) and maximal viscosity (*η*) decreased by 32–65% at 100 μM fatty acid concentrations as shown in Table 2 and Fig 6.

**Table 2:** Effect of free fatty acids on the viscoelasticity of fibrin clots.

	G’ (Pa)	G” (Pa)	loss tangent	*η* (kPaˑs)	*τ*_*flow*_ (Pa)
**no additive**	73.1 (1.6)	7.9 (0.2)	0.1 (0.004)	10.9 (0.8)	522 (46)
**oleate**	36.7* (4.5)	3.8* (0.5)	0.1 (0.001)	3.9* (1.1)	353* (31)
**stearate**	46.5* (7.5)	5.4* (0.8)	0.1 (0.003)	3.8* (1.5)	346* (74)

Mean values for storage modulus (G’), loss modulus (G”), their ratio (G”/G’, loss tangent) after 10 min clotting, maximal viscosity values (η) and critical shear stress (τ_flow_) values are presented (with standard deviations).

* Asterisk indicates *p*<0.05 according to the Kolmogorov-Smirnov test in comparison to pure fibrin, *n* = 4.

## Discussion

Despite many advances in diagnosis and management, complications of arterial embolism are still a leading cause of disability and death. Most arterial emboli originate in the left heart, manifesting mainly in embolic stroke or transient ischemic attack. The second most common cause of morbidity and mortality from arterial embolic disease is limb ischemia in the lower extremities. Less frequently, emboli target the upper extremities, mesenteric or renal arteries [38]. Embolization may be a consequence of mechanical instability of the fibrin clot in the subacute phase of an arterial occlusion or as a response to the thrombolytic or invasive endovascular intervention [39–41]. Thus, during the treatment of arterial thrombosis at different locations (acute ischemic stroke, myocardial infarction or peripheral arterial occlusion) the possibility of embolization has to be considered as a crucial determinant of the therapeutic outcome. The mechanical stability of an early thrombus is the overall consequence of the presence and actual local concentration of the molecular and cellular participants of clot formation. Therefore, different genetic, environmental or acute pathophysiological factors may lead to altered clot structure and stability [7,8,28,31]. During the process of arterial thrombus formation, free fatty acids are released from activated platelets and therefore emerge as potential modulators of fibrin clot formation. Our present study addressed the impact of free fatty acids on thrombin mediated fibrin generation and the mechanical properties of the formed clot.

The bell shape of the response in clotting time to the applied dose of fatty acid (Fig 1) suggests a physical interaction between thrombin and the fatty acid in the form of a ternary complex, in which fibrinogen or fibrin monomer also participates. In this ternary complex, the modulator fatty acid exerts a kinetic template effect by promoting the favorable spatial organization for the inhibition of the enzyme reaction or modification of the polymerization process. Maximal alteration occurs at an optimal concentration ratio of the three components [42]. An analogous ternary complex has been reported previously consisting of thrombin, fibrin monomer and heparin, the formation of which altered the activity of thrombin on its different substrates [43]. Due to the known resistance of thrombin to autolysis [44], the kinetics of thrombin autodigestion is rather slow to account for the observed inhibition of clotting, though its acceleration in the presence of fatty acids suggests molecular interactions between fatty acids and thrombin even in the absence of fibrin(ogen). This physical interaction affects the action of thrombin on low-molecular weight peptide substrates, too. Both fatty acids altered the thrombin activity on Boc-VPR-AMC, but with different kinetic patterns: 200 μM oleate caused a marked, 8-fold increase in *K*_*m*_, while both fatty acids resulted in a minor drop in the catalytic constant.

Dynamic light scattering data indicate free fatty acids form aggregates under our experimental conditions and aggregated long chain fatty acids are likely to form hydrogen bonds with fibrinogen and thrombin in the proposed ternary complex through carboxylic groups. Hydrophobic interactions between a few alkyl chains (which may be available on the surface of the fatty acid aggregate) and hydrophobic regions of fibrinogen and the active site and/or Exosite I of thrombin are also conceivable [45,46]. Differences in the effects of stearic acid and oleic acid could be traced back to known differences in their aggregate assembly. Because of the kink in the aliphatic chain at the *cis*-double bond, oleic acid forms loose aggregates with 50% greater intermolecular distance between the polar groups on their surface than stearic acid [33], which could also result in a different steric assembly of the ternary complex.

The ultimate structure and mechanical properties of fibrin depend on the relative kinetics of two sequential steps in its formation: 1) catalytic action of thrombin on fibrinogen, and 2) polymerization of the formed fibrin monomers. Each of these steps could be independently targeted by modulators of fibrin formation. In most cases a decrease in thrombin activity leads to a coarser fibrin network with less frequently branched thicker fibers consisting of more protofibrils [34], but incorporation of non-fibrin molecules into the fibrin meshwork modifies the fiber diameter independently of the thrombin concentration, e.g. DNA increases [31], whereas heparin decreases fiber size [47] without any direct effect on thrombin activity. The marked prolongations in clotting times, as well as the major structural-mechanical changes in the presence of both stearate and oleate are clearly disproportionate with the minor changes in the *k*_*p*_ values in our enzyme kinetic measurements using a small synthetic peptide substrate. Further discrepancy is the stearate increasing fibrin fiber diameters, which could alone easily be explained by lower thrombin activities and prolonged clotting times, however we found no fibrin fiber thickening in the presence of oleate, despite a marked increase in *K*_*m*_ for the fluorogenic substrate and a delay in clotting. Thus, such disproportionate structural changes might be attributed mainly to alterations in the polymerization step of fibrin formation and to a lesser extent to effects through thrombin activity. The maximal prolongation of clotting times by the fatty acids could be explained by a subtle delay in fibrin polymerization due to the presence of fibrin(ogen)-thrombin-fatty acid ternary complexes, and potentially fibrinogen-fatty acid aggregates, which -in the case of stearate- could incorporate into fibrin bundles, and appear as fibrin fiber thickening. A similar phenomenon has been reported by Yeromonahos et al., observing increased fibrin porosity and fiber diameters due to incorporation of thrombin-fibrin monomer-unfractionated heparin ternary complexes into the fibers [48].

Despite the differences in fiber diameters, the mechanical properties of the clots formed in the presence of the two fatty acids were rather similar according to the viscoelastic parameters gained by rheometry. A mechanically less stable fibrin network is suggested by the diminished values of the storage and loss moduli (G’ and G”). The loss tangent (G”/G‘), however, remained unchanged. Data in the literature [49] suggest that the formation of thicker fibers consisting of more protofibrils due to lower thrombin activity result in increased clot rigidity (G’). Moreover, since these clots usually also present with less frequent branching, these thicker fibers tend to suffer a higher degree of intrafiber rearrangement upon deformation, which results in a higher energy loss observed as a higher loss tangent value [49]. The rheological effects of fatty acids in our experiments are commensurate with a different mechanism, which also argues indirectly against the possibility of fatty acids inhibiting thrombin activity on fibrinogen. Thinner fibers, higher clot deformability (decreased G' and G") and an unchanged loss tangent have been described in fibrin structures formed at decreasing fibrinogen concentrations [49], which suggests that the presence of fatty acids may result in less effectively polymerizing fibrin monomers. In the case of oleate, thinner fibers were formed (similar to the effect observed at a lower fibrinogen concentration), while incorporation of the abovementioned ternary complexes overcomes this effect when stearate is present during the clotting phase. The dynamic viscosity (η_max_) of fibrin was also lowered in the presence of fatty acids indicating decreased internal resistance. Moreover, according to the τ_flow_ values, less energy was necessary to reach the gel-fluid transition, the point, at which the fibrin matrix lost its physical integrity. Thus, mechanically less stable and more deformable clots were formed, when free fatty acids were present at physiological concentrations during fibrin polymerization. If we compare the shear stress values reported for partially occluded blood vessels (about 230 Pa at 80% occlusion of coronary arteries) [27] and the shear stress needed to reach the gel-fluid transition in our experimental setup, it can be concluded that stearic and oleic acid decrease the critical shear stress necessary for disassembly of fibrin to values that are likely to act on *in vivo* thrombi.

## Conclusions and Perspectives

Since fibrin structure is a major determinant of the mechanical stability and lytic susceptibility of thrombi [36,50], we can conclude that free fatty acids at their physiological concentrations may destabilize intravascular thrombi. These *in vitro* findings prompt further investigations on the free fatty acid content of embolizing *ex vivo* thrombi so that predictive conclusions for prevention of *in vivo* microembolization could be drawn in the clinical practice.

## References

[1] Pancak J, Wagnerova H, Szárazová AŠ, Blaho A, Durovsky O, Durovska J. Multi-infarct dementia and Alzheimer disease, contribution of cerebral circulation ultrasonography to pathogenesis and differential diagnosis. Value of microembolisation. Neuro Endocrinol Lett. 2016; 37:137–140. 27179577

[2] Idicula T, Thomassen L. Microemboli Monitoring in Ischemic Stroke In: Acute Ischemic Stroke. In Tech; 2012 pp. 145–156.

[3] Man BL, Fu YP, Chan YY, Lam W, Hui AC, Leung WH, et al Lesion patterns and stroke mechanisms in concurrent atherosclerosis of intracranial and extracranial vessels. Stroke. 2009; 40:3211–3215. 10.1161/STROKEAHA.109.557041 19644065

[4] Schwartz RS, Burke A, Farb A, Kaye D, Lesser JR, Henry TD, et al Microemboli and microvascular obstruction in acute coronary thrombosis and sudden coronary death: relation to epicardial plaque histopathology. J Am Coll Cardiol 2009; 54: 2167–2173. 10.1016/j.jacc.2009.07.042 19942088

[5] Yamada T, Yoshii T, Yoshimura H, Suzuki K, Okawa A. Upper limb amputation due to a brachial arterial embolism associated with a superior mesenteric arterial embolism: a case report. BMC Res Notes 2012; 5: 372 10.1186/1756-0500-5-372 22828325 PMC3410779

[6] Dag O, Kaygın MA, Erkut B. Analysis of Risk Factors for Amputation in 822 Cases with Acute Arterial Emboli. Scientific World Journal 2012; 2012:673483 10.1100/2012/673483 22606056 PMC3346294

[7] Wolberg AS. Plasma and cellular contributions to fibrin network formation, structure and stability. Haemophilia 2010; 16 Suppl 3: 7–12. 10.1111/j.1365-2516.2010.02253.x 20586795

[8] Undas A, Ariëns RA. Fibrin clot structure and function: a role in the pathophysiology of arterial and venous thromboembolic diseases. Arterioscler Thromb Vasc Biol 2011; 31: e88–99. 10.1161/ATVBAHA.111.230631 21836064

[9] Burke JE, Dennis EA. Phospholipase A2 structure/function, mechanism, and signaling. J Lip Res 2009; 50(Suppl):S237–S242. 10.1194/jlr.R800033-JLR200 PMC2674709 19011112

[10] McBane RD 2nd, Ford MA, Karnicki K, Stewart M, Owen WG. Fibrinogen, fibrin and crosslinking in aging arterial thrombi. Thromb Haemost 2000; 84: 83–87. 10928475

[11] Slatter DA, Aldrovandi M, O'Connor A, Allen SM, Brasher CJ, Murphy RC, et al Mapping the Human Platelet Lipidome Reveals cytosolic phospholipase A_2_ as a regulator of mitochondrial bioenergetics during activation. Cell Metab 2016; 23: 930–944. 10.1016/j.cmet.2016.04.001 27133131 PMC4873619

[12] Rábai G, Váradi B, Longstaff C, Sótonyi P, Kristóf V, Tímár F, et al Fibrinolysis in a lipid environment: modulation through release of free fatty acids. J Thromb Haemost 2007; 5: 1265–1273. 10.1111/j.1538-7836.2007.02556.x 17403096 PMC1974781

[13] Marcus AJ, Ullman HL, Safier LB. Lipid composition of subcellular particles of human blood platelets. J Lip Res 1969; 10: 108–114. 5764109

[14] Fraser DA, Thoen J, Rustan AC, Førre O, Kjeldsen-Kragh J. Changes in plasma free fatty acid concentrations in rheumatoid arthritis patients during fasting and their effects upon T-lymphocyte proliferation. Rheumatology (Oxford). 1999; 38: 948–952. 10534544 10.1093/rheumatology/38.10.948

[15] Tanka-Salamon A, Tenekedjiev K, Machovich R, Kolev K. Suppressed catalytic efficiency of plasmin in the presence of long-chain fatty acids. Identification of kinetic parameters from continuous enzymatic assay with Monte Carlo simulation. FEBS J 2008; 275: 1274–1282. 10.1111/j.1742-4658.2008.06288.x 18279394 PMC2447916

[16] Higazi AAR, Aziza R, Samara AA, Mayer M. Regulation of fibrinolysis by non-esterified fatty acids. Biochem J 1994; 300: 251–255. 8198542 10.1042/bj3000251 PMC1138149

[17] Vanschoonbeek K, Feijge MA, Paquay M, Rosing J, Saris W, Kluft C, et al Variable hypocoagulant effect of fish oil intake in humans: modulation of fibrinogen level and thrombin generation. Arterioscler Thromb Vasc Biol 2004; 24: 1734–1740. 10.1161/01.ATV.0000137119.28893.0b 15217806

[18] Larson MK, Tormoen GW, Weaver LJ, Luepke KJ, Patel IA, Hjelmen CE, et al Exogenous modification of platelet membranes with the omega-3 fatty acids EPA and DHA reduces platelet procoagulant activity and thrombus formation. Am J Physiol Cell Physiol 2013; 304: C273–729. 10.1152/ajpcell.00174.2012 23174566 PMC3566437

[19] Phang M, Scorgie FE, Seldon M, Garg ML, Lincz LF. Reduction of prothrombin and Factor V levels following supplementation with omega-3 fatty acids is sex dependent: a randomised controlled study. J Nutr Biochem 2014; 25: 997–1002. 10.1016/j.jnutbio.2014.05.001 24997005

[20] Melzig MF, Henke K. Inhibition of thrombin activity by selected natural products in comparison to neutrophil elastase. Planta Med 2005; 71: 787–789. 10.1055/s-2005-871253 16142650

[21] Viskupicova J, Danihelova M, Majekova M, Liptaj T, Sturdik E. Polyphenol fatty acid esters as serine protease inhibitors: a quantum-chemical QSAR analysis. J Enzyme Inhib Med Chem 2012; 27: 800–809. 10.3109/14756366.2010.616860 21981000

[22] Grütter MG, Priestle JP, Rahuel J, Grossenbacher H, Bode W, Hofsteenge J, et al Crystal structure of the thrombin-hirudin complex: a novel mode of serine protease inhibition. EMBO J 1990; 9: 2361–2365. 2369893 10.1002/j.1460-2075.1990.tb07410.x PMC552259

[23] Fuentes-Prior P, Noeske-Jungblut C, Donner P, Schleuning W-D, Huber R, Bode W. Structure of the thrombin complex with triabin, a lipocalin-like exosite-binding inhibitor derived from a triatomine bug. Proc Natl Acad Sci U S A 1997; 94: 11845–11850. 9342325 10.1073/pnas.94.22.11845 PMC23629

[24] Van de Locht A, Lamba D, Bauer M, Huber R, Friedrich T, Kröger B, et al Two heads are better than one: crystal structure of the insect derived double domain Kazal inhibitor rhodniin in complex with thrombin. EMBO J 1995; 14: 5149–5157. 7489704 10.1002/j.1460-2075.1995.tb00199.x PMC394622

[25] Tucker TJ, Brady SF, Lumma WC, Lewis SD, Gardell SJ, Naylor-Olsen AM, et al Design and synthesis of a series of potent and orally bioavailable noncovalent thrombin inhibitors that utilize nonbasic groups in the P1 position. J Med Chem 1998; 41: 3210–3219. 10.1021/jm9801713 9703466

[26] Scheraga HA. The thrombin-fibrinogen interaction. Biophys Chem 2004; 112: 117–130. 10.1016/j.bpc.2004.07.011 15572239

[27] Maalej N, Folts JD. Increased shear stress overcomes the antithrombotic platelet inhibitory effect of aspirin in stenosed dog coronary arteries. Circulation 1996; 93: 1201–1205. 8653842 10.1161/01.cir.93.6.1201

[28] Varjú I, Sótonyi P, Machovich R, Szabó L, Tenekedjiev K, Silva MM, et al Hindered dissolution of fibrin formed under mechanical stress. J Thromb Haemost 2011; 9: 979–986. 10.1111/j.1538-7836.2011.04203.x 21251205 PMC3093023

[29] Lundblad RL, Kingdon HS, Mann KG. Thrombin. Methods Enzymol 1976; 45: 156–176. 1011989 10.1016/s0076-6879(76)45017-6

[30] Longstaff C, Wong MY, Gaffney PJ. An international collaborative study to investigate standardisation of hirudin potency. Thromb Haemost 1993; 69: 430–435. 8322265

[31] Longstaff C, Varjú I, Sótonyi P, Szabó L, Krumrey M, Hoell A, et al: Mechanical Stability and Fibrinolytic Resistance of Clots Containing Fibrin, DNA, and Histones. J Biol Chem 2013; 288: 6946–6956. 10.1074/jbc.M112.404301 23293023 PMC3591605

[32] Nikolova ND, Toneva-Zheynova D, Kolev K, Tenekedjiev K. Monte Carlo statistical tests for identity of theoretical and empirical distributions of experimental data In: Theory and applications of Monte Carlo simulations. InTech 2013; pp. 1–26.

[33] Kanicky JR, Shah DO. Effect of degree, type, and position of unsaturation on the pKa of long-chain fatty acids. J Colloid Interface Sci 2002; 256: 201–207. 12505514 10.1006/jcis.2001.8009

[34] Longstaff C, Thelwell C, Williams SC, Silva MM, Szabó L, Kolev K. The interplay between tissue plasminogen activator domains and fibrin structures in the regulation of fibrinolysis: kinetic and microscopic studies. Blood 2011; 117: 661–668. 10.1182/blood-2010-06-290338 20966169 PMC3031486

[35] Varjú I, Longstaff C, Szabó L, Farkas ÁZ, Varga-Szabó VJ, Tanka-Salamon A, et al DNA, histones and neutrophil extracellular traps exert anti-fibrinolytic effects in a plasma environment. Thromb Haemost 2015; 113: 1289–1298. 10.1160/TH14-08-0669 25789443

[36] Weisel JW, Litvinov RI. The biochemical and physical process of fibrinolysis and effects of clot structure and stability on the lysis rate. Curr Med Chem-Cardiovasc Hematol Agents 2008; 6: 161–180. 10.2174/187152508784871963 18673231

[37] Longstaff C, Kolev K. Basic mechanisms and regulation of fibrinolysis. J Thromb Haemost 2015; 13 Suppl 1: S98–105. 26149056 10.1111/jth.12935

[38] Lyaker MR, Tulman DB, Dimitrova GT, Pin RH, Papadimos TJ. Arterial embolism. Int J Crit Illn Inj Sci 2013; 3:77–87. 10.4103/2229-5151.109429 23724391 PMC3665125

[39] Sila C. Neurologic complications of cardiac tests and procedures. Handb Clin Neurol. 2014; 119:41–47. 10.1016/B978-0-7020-4086-3.00004-7 24365287

[40] Stanek F, Ouhrabkova R, Prochazka D. Mechanical thrombectomy using the Rotarex catheter in the treatment of acute and subacute occlusions of peripheral arteries: immedite results, long-term follow-up. Int Angiol. 2013; 32:52–60. 23435392

[41] Zhou ZG, Wang RL, Yu KL. Myocardial infarction following recombinant tissue plasminogen activator treatment for acute ischemic stroke: a dangerous complication. Chin Med J (Engl). 2012; 125:2775–2776. 22931992

[42] Douglass EF, Miller CJ, Sparer G, Shapiro H, Spiegel DA. A Comprehensive Mathematical Model for Three-Body Binding Equilibria. J Am Chem Soc 2013; 135:6092–6099. 10.1021/ja311795d 23544844 PMC3717292

[43] Hogg PJ, Jackson CM. Formation of a ternary complex between thrombin, fibrin monomer, and heparin influences the action of thrombin on its substrates. J Biol Chem 1990; 265: 248–255. 2294105

[44] Chang JY. The structures and proteolytic specificities of autolysed human thrombin. Biochem J 1986; 240:797–802. 3827867 10.1042/bj2400797 PMC1147489

[45] Scarsi M, Majeux N, Caflisch A. Hydrophobicity at the surface of proteins. Proteins 1999; 37: 565–575. 10651272 10.1002/(sici)1097-0134(19991201)37:4<565::aid-prot7>3.0.co;2-v

[46] Fuentes-Prior P, Iwanaga Y, Huber R, Pagila R, Rumennik G, Seto M, et al Structural basis for the anticoagulant activity of the thrombin-thrombomodulin complex. Nature 2000; 404: 518–525. 10.1038/35006683 10761923

[47] Longstaff C, Hogwood J, Gray E, Komorowicz E, Varjú I, Varga Z, et al Neutralisation of the anti-coagulant effects of heparin by histones in blood plasma and purified systems. Thromb Haemost 2016; 115: 591–599. 10.1160/TH15-03-0214 26632486

[48] Yeromonahos C, Marlu R, Polack B, Caton F. Antithrombin-independent effects of heparins on fibrin clot nanostructure. Arterioscler Thromb Vasc Biol. 2012; 32:1320–1324. 10.1161/ATVBAHA.112.245308 22362760

[49] Ryan EA, Mockros LF, Weisel JW, Lorand L. Structural origins of fibrin clot rheology. Biophys J 1999; 77: 2813–2826. 10.1016/S0006-3495(99)77113-4 10545379 PMC1300553

[50] Weisel JW. Structure of fibrin: impact on clot stability. J Thromb Haemost. 2007; 5(Suppl 1):116–124. 17635717 10.1111/j.1538-7836.2007.02504.x

